# Review of Supplements That Patients Commonly Report Using for Dementia

**DOI:** 10.3390/jcm13247541

**Published:** 2024-12-11

**Authors:** Alexander Frolov, Audrey Wadood, Brendan J. Kelley

**Affiliations:** Department of Neurology, University of Texas Southwestern Medical Center, Dallas, TX 75390, USA; alexander.frolov@utsouthwestern.edu (A.F.);

**Keywords:** dementia, alternative medicine, supplements, brain health

## Abstract

Dietary supplements are readily available over the counter in the United States and are used by the majority of older adults to address a variety of concerns and conditions. Many older adults report using dietary supplements for cognitive health—either to address memory loss or dementia or in efforts to prevent cognitive decline. Our objective for this narrative review is to summarize the available efficacy and safety data for several supplements commonly reported in our clinic as being used for symptoms of dementia. Using a validated survey instrument, we conducted a survey of patients in our tertiary referral center memory clinic population to assess for the most commonly reported supplements for cognition. In our review, we compare the strength of published medical and scientific evidence to advertising or other lay press claims made about the nine most reported supplements with the aim of providing a representation of general trends in this industry. We found little or no scientific evidence available to support the use of any of these substances to ameliorate memory loss or other cognitive symptoms. Although most appear safe in the studies conducted to date, several authors have highlighted the lack of Food and Drug Administration oversight in the supplement industry, raising concerns over unknown or undeclared contaminants in these over-the-counter products. This review will better prepare clinicians to discuss these considerations with their patients who are considering the use of dietary supplements.

## 1. Introduction

Neurodegenerative disorders, of which Alzheimer’s disease is the most common, are progressive and debilitating conditions with historically limited treatment options. Only recently were two disease-modifying treatments approved by the Food and Drug Administration (FDA) for use in people with mild cognitive impairment and mild dementia due to Alzheimer’s, though these treatments do not cure or fully arrest the disease, and no medication has demonstrated a preventive effect. Patients with Alzheimer’s disease face a prognosis of progressive cognitive decline and loss of independence. Older adults, many of whom have seen the impact of this disease on a family member or friend, face the prospect of developing dementia, a condition whose dominant risk factor is advanced age. In this milieu, it is no surprise that a large and continually growing market for unregulated alternative treatments in the realm of vitamins, herbs, “medical foods”, and other supplements has emerged.

US consumers spent more than USD 95 billion on nutritional supplements in 2023, representing 23% of the global expenditure of roughly USD 414 billion, according to a study by Grand View Research, a U.S.-based research and marketing firm [1]. While this estimate includes “functional foods and beverages”, a segment that accounts for expenditure on probiotics and the larger markets of sports drinks and “enhanced” water, other sources estimate U.S. expenditure on nutritional supplements in 2023 to be between USD 45 and USD 56 billion [2], which aligns with the 2012 National Center for Complementary and Integrative Health survey data, which estimated the expenditure on complementary health to be USD 30 billion [3].

Much of the growth in supplement and vitamin use is driven by perceived nutritional or dietary deficiencies and beliefs that supplements aid in the treatment or prevention of aging-related health conditions including heart disease, dyslipidemia, alopecia, and cognitive decline. The use of supplements among adults in the U.S. has steadily increased for at least the past 20 years, and these reports project a compound annual growth rate between 4.8 and 8.9% from 2024 to 2034.

These societal observations certainly extend to older adults. The National Health and Nutrition Examination Survey (NHANES) study reported 70% of adults aged 60 years and older take a regular dietary supplement, with 29% reporting using four or more daily [4]. This result is consistent with the Health and Retirement Study (HRS), in which 84.6% of participants reported the regular use of dietary supplements, taking a mean of 3.2 different dietary supplements and 41.9% of the HRS participants reported taking four or more supplements daily [5]. A survey of participants in the ASPREE (ASPirin in Reducing Events in the Elderly) trial which enrolled people over age 70 in the U.S. and Australia reported that two-thirds of the participants regularly used supplements [6]. This high rate of supplement use is particularly concerning when considered in light of a 2019 AARP survey in which half of adults over age 50 falsely believed that the FDA determines dietary supplements to be both safe and effective before they are sold [7,8].

These data suggest that many consumers have a false sense of safety and legitimacy for claims made by supplement advertisers. The aggressive direct-to-consumer marketing of supplements commonly overstates health benefits, with the onus resting on states and consumers to disprove false claims. A recent notable example is the ruling against Prevagen manufacturer Quincy Bioscience for having made fraudulent and deceptive statements about a supplement and violating New York’s consumer protection laws [9]. That this ruling is notable underscores the reality that many similarly deceptive tactics likely go unchallenged.

As a category, supplements and other alternative treatments marketed for neurodegenerative disorders specifically offer a trifecta of features that at least partially explain their popularity. Firstly, these substances are sold and marketed as supplements or “medical foods” that do not require rigorous clinical trials or regulatory approval or oversight. Companies are thus able to make exaggerated or incomplete statements regarding their efficacy and side effects, the end result of which is often the implied promise of cognitive recovery or disease slowing in conditions for which no such treatment has been found. Secondly, these substances can be sold over the counter and without a prescription, providing patients easy access and autonomy without need for costly medical evaluations or prescriptions. And, thirdly, supplements are often marketed as “all natural”, “naturally derived”, or non-pharmaceutical substances, implying they represent a healthier alternative to prescription medications. This last factor also plays to consumer concerns of pharmaceutical side effects and a false belief that “natural” remedies are devoid of similar adverse effects.

This review will provide an updated summary of several commonly used and marketed supplements for dementia and brain health, focusing on the science underpinning claims of efficacy. The chosen supplements are not all-inclusive but are meant to be representative of the general trends in the supplements-for-cognition industry.

## 2. Methods

**Figure 1:**
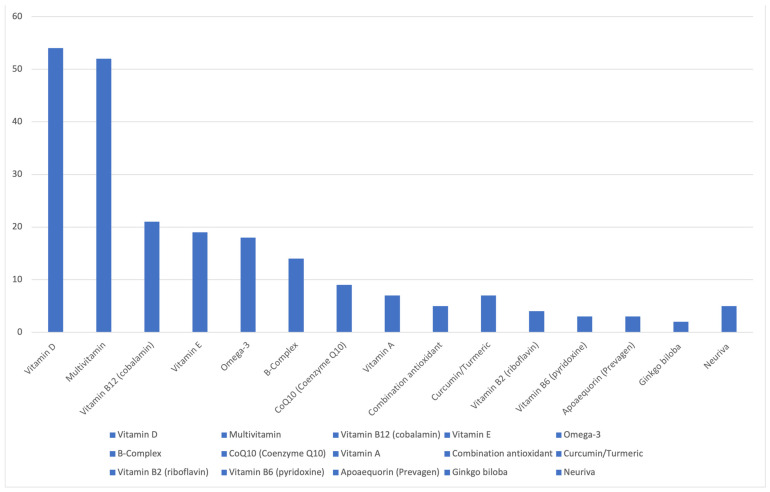
Frequency of reported supplements.

In order to determine what supplements to focus on for this review, a survey was conducted in the authors’ clinics in the Cognition and Memory Disorders Clinic at UT Southwestern Medical Center (Dallas, TX, USA). All new patients arriving to the clinic were offered to voluntarily participate in the survey. The survey design was based on a validated instrument designed to assess intake of dietary supplements and adapted for the purpose of assessing supplement use pertinent to memory disorders [10]. One hundred eighteen surveys were ultimately collected and reviewed. Multiple supplements were reported (Figure 1), many of which were being used for various non-cognitive indications. Based on the survey results and the authors’ clinical experience, nine supplements were chosen for in-depth review. Table 1 provides a summary of the reviewed supplements and the available data.

This study was conducted in accordance with the guidelines of the Declaration of Helsinki and approved by the Institutional Review Board of The University of Texas Southwestern Medical Center (STU-2022-0012; approved 2 January 2022).

## 3. Review of Several Supplements Patients Commonly Reported Using for Cognition

### 3.1. Apoaequorin (Prevagen^®^)

In recent years, Prevagen^®^ (Quincy Bioscience, Middleton, WI, USA) has become one of the most commonly mentioned supplements for memory loss by patients in the authors’ clinics, perhaps secondary to focused advertising efforts. In 2021, an AARP, formerly American Association of Retired Persons, survey found that Prevagen^®^ accounted for 4% of respondents taking supplements for brain health [57].

#### 3.1.1. Purported Mechanism of Action

Apoaequorin is a protein initially discovered in *Aequorea victoria* jellyfish. It is a calcium-binding component of aequorin, by which jellyfish use calcium as a trigger for oxidation of a light-emitting molecule in aequorin [58]. Excessive calcium influx into cells can lead to excitotoxic cell death during ischemia and other states of oxidative stress, and calcium-binding proteins can help regulate intracellular calcium levels and prevent cell death [12]. Furthermore, basic science studies have shown calcium signaling is important in memory consolidation. Based on these observations, it was hypothesized that apoaequorin might protect hippocampal cells from calcium-based excitotoxic injury, thereby protecting or improving memory. Prevagen^®^ is a synthetic formulation of apoaequorin produced by Quincy Bioscience Manufacturing, Inc. The company claims that Prevagen^®^ is designed for healthy, non-demented individuals experiencing normal age-related cognitive changes rather than those with significant, pathologic cognitive impairment.

#### 3.1.2. Available Study Data

In a pre-clinical study funded by Quincy Neuroscience, Detert et al. infused apoaequorin into rat hippocampi, after which hippocampal slices were prepared and subjected to five minutes of oxygen and glucose deprivation, and resultant cell death was assayed using trypan blue staining [12]. Slices from hippocampi pre-treated with apoaequorin showed significantly fewer dead cells as compared to controls, and this effect was dose dependent. The study authors concluded that apoaequorin may have neuroprotective properties. Other in vitro and in vivo animal studies by the same study group were reported to be concordant [59].

In a clinical study funded by Quincy Neuroscience, a double-blind, placebo-controlled design investigated the clinical effects of apoaequorin [11]. A total of 218 participants with self-reported cognitive concerns aged 40–95 were randomized in a 3:2 ratio to either 10 mg of daily apoaequorin or placebo and treated for up to 90 days. Analysis included a baseline self-report of cognitive function (AD8 screening tool) as well an adapted computerized cognitive assessment (CogState Research Battery) measuring verbal learning, memory, executive function, psychomotor function, attention, visual learning, and working memory. The primary endpoint was a change in cognitive testing from baseline to day 90. There were no statistically significant results across the study population. A post-hoc subgroup analysis of 97 participants with mild subjective cognitive impairments at the start of the study (low scores on the AD8 screening tool), a statistically significant increase in verbal learning and memory was demonstrated in the intervention but not in the placebo group (i.e., placebo group improved by 3.79% while intervention group improved by 10.86% in verbal learning, and placebo improved by 7.41% while intervention group improved by 15.82% in delayed recall). Even if the statistics are taken at face value, the clinical significance would be questionable (i.e., a 10% improvement in a 12-item list learning task would amount to approximately one extra learned word).

Post-publication commentary highlighted methodological issues with validity related to the lack of data regarding drug absorption, the high likelihood of apoaequorin being hydrolyzed by gut proteases, and the inability of apoaequorin to effectively cross the blood–brain barrier [58].

#### 3.1.3. Safety Data and Side Effects

During the clinical trial conducted by the drug manufacturer discussed above, there were no serious adverse events. One participant in the intervention arm withdrew due to feeling irritable, and another participant in the placebo arm withdrew due to feeling despondent and tired, but neither sought further medical care for these symptoms. Previous rat studies showed no adverse effects [60].

#### 3.1.4. Summary

There are no reliable data of clinical efficacy for Prevagen^®^ in any human condition. The pre-clinical in vitro data show mechanistically plausible effects; however, the only published clinical trial did not meet its primary endpoint of demonstrating benefit at 90 days. Although a subgroup analysis of this small trial suggested possible benefit, this finding has not been followed up with publication of a prospective investigation in this population. There are also legitimate questions as to the pharmacokinetics and absorption with oral administration. While the supplement is purportedly marketed to those with normal cognitive function or mild cognitive deficits, this information is relegated to a footnote on the company’s website (www.prevagen.com, accessed 9 July 2024) [61], and advertising language in general suggests a clinically significant benefit in cognition from the supplement. In the authors’ experience, patients often understand the advertising to imply Prevagen as a treatment for Alzheimer’s-related cognitive impairment, though this has never been studied directly. Indeed, this likely contributed to a jury trial verdict against Quincy Bioscience “for deceptive and fraudulent advertising”, though in our experience, patients remain frequently misinformed on this topic [9]. Finally, the cost of the supplement could be significant for retirees who are living on fixed incomes.

### 3.2. Gingko Biloba

The Ginkgo biloba tree likely has its origins in China and is thought to be one of the oldest living tree species. It is now cultivated in many parts of the world, can survive for 1000 years, and is resistant to insects, fungi, and frosts [29]. Various parts of the tree have a long history as herbal supplements used for many different indications, including neurodegenerative disorders, peripheral vascular disease, myocardial infarction, stroke, and tinnitus, among others. Historic medicinal uses are documented as long as 5000 years ago in China where it was used as a treatment for various disorders such as asthma [62,63].

#### 3.2.1. Pharmacology and Purported Mechanism of Action

Various preparations of the Ginkgo biloba tree have been used over time. In the modern era, the first and most commonly used standardized extract (EGb 761) of Ginkgo biloba was created by a German pharmaceutical company and contains 6% terpene lactones (ginkgolides and bilobalide) and 24% flavonoids, which are thought to be the active ingredients [64]. Some of the terpenoids (the ginkgolides) have been shown to act as platelet-activating factor antagonists, thereby inhibiting platelet activation. Flavonoids are known to be antioxidants and can also act as heavy metal chelators. These antiplatelet and antioxidant effects underpin the purported benefit in various vascular and inflammatory conditions. A reduction in ABeta-related neurotoxicity has also been suggested in Alzheimer’s disease [29,30,34].

#### 3.2.2. Efficacy Data

Much preclinical data regarding the various effects of Gingko biloba extract (GBE) exist, with most studies using the EGb 761 extract. In vitro studies have demonstrated protection of neurons against various insults, including hypoxia [25], hydrogen peroxide [23], glutamate, verapamil [24], beta-amyloid [26,27,28,65], and others [66]. In vivo animal studies have also documented reductions in neuronal injury after transient ischemia [67] and other hypoxic stressors. Multiple preclinical studies have demonstrated various mechanisms for such neuroprotection, including platelet-aggregation-related increases in blood flow [68], antioxidant effects [32], and other factors including effects on various neurotransmitter systems [28]. There is evidence for GBE’s role as an antioxidant [33,35] anti-inflammatory [2,33], stress-reducing [21,31], and neuro-protective substance [21,22,31,69,70,71].

Regarding clinical efficacy in cognitive disorders, the data are mixed and overall do not support clinical effectiveness. A 2009 Cochrane review [20] evaluated 36 randomized, double-blind studies, with most employing the EGb 761 extract at various doses. The trials overall showed inconsistent results, with a few smaller, older, and less reliable studies suggesting benefit, but larger, more reliable, and more recent studies failing to corroborate this. Three out of four of the most recent studies in the review showed no difference between GBE and placebo, while one showed a treatment effect in favor of GBE. A subgroup analysis of only patients with Alzheimer’s disease in the same Cochrane review included 925 patients from nine trials and showed “no consistent pattern of any benefit associated with Ginkgo biloba”. The review’s conclusion is that “there is no convincing evidence that Ginkgo biloba is efficacious for dementia and cognitive impairment”.

A well-structured, double-blind clinical trial that enrolled 513 outpatients with uncomplicated Alzheimer’s dementia evaluated 26-week treatment with GBE at daily doses of 120 mg or 240 mg or placebo [13]. The authors reported no significant between-group differences on measures of cognitive and functional decline. They did note that the subgroup of patients on GBE with neuropsychiatric symptoms showed significantly better cognitive performance and global assessment scores.

Research into GBE’s potential benefits in cognitive disorders has remained robust since publication of the 2009 Cochrane review. One well-structured trial investigating the potential of a well-characterized ginkgo extract to prevent dementia was the Ginkgo Evaluation of Memory (GEM) study [17]. This randomized, double-blind, placebo-controlled clinical trial enrolled 3069 community-dwelling adults aged 72 to 96 years and reported findings based upon a median follow-up of 6.1 years. The authors found no difference in cognitive decline compared to the placebo group.

A 2012 clinical trial (GuidAge) [14] again evaluated GBE for prevention of Alzheimer’s disease. This parallel-group, double-blind, placebo-controlled trial enrolled 2854 non-demented participants followed for at least five years. The study did not demonstrate any statistically significant difference between the GBE and placebo groups in rates of new Alzheimer’s disease diagnosis (1.2 cases per 100 person-years in the GBE group and 1.4 cases per 100-person years in the placebo group, for a hazard ratio of 0.84, CI 0.60–1.18, and *p* = 0.306). An earlier randomized controlled trial for prevention with median follow-up of 6.1 years showed similarly negative results [16].

Most other clinical studies were of lower quality with higher potential for bias and/or methodological shortcomings, such as non-blinded, smaller, or shorter studies [15,18,72]. Some have suggested potential clinical benefit, though this has not been replicated in larger, longer, placebo-controlled trials.

GBE has otherwise been evaluated as a treatment for cognition in various other contexts, for example, in cerebrovascular disease. A randomized controlled trial assessing cognitive decline after stroke which reported a modest statistically significant decrease in rate of cognitive decline in patients treated with GBE as compared to placebo at six months—namely a difference of 1.29 points in Montreal Cognitive Assessment (MoCA) scores at six months [19]. The clinical meaningfulness or durability of these findings is unclear and has not been replicated.

#### 3.2.3. Safety Data and Side Effects

Despite its purported anti-coagulant and anti-platelet effect, the studies discussed above did not find an increased incidence of bleeding risk or other adverse effects in GBE as compared to placebo [20].

#### 3.2.4. Summary

Ginkgo biloba has an extensive history of use for a variety of ailments. Unlike Prevagen, there is robust good-quality literature regarding the use of GBE for cognitive decline. The highest-quality clinical trial data on GBE’s use have not demonstrated efficacy in treatment or prevention of cognitive disorders. Smaller trials at times show benefits that are not clearly clinically significant and are not replicated in larger trials. GBE does appear to be safe, though a theoretical risk could exist for patients who are already on anticoagulants or those who have clotting or bleeding disorders (something especially pertinent in the age of Alzheimer’s disease anti-amyloid monoclonal antibody therapy, for which a risk of intracranial bleeding exists). Its cost is relatively low. Based upon the available efficacy and safety data, and the subgroup analysis in the GEM trial, it may have a role for those with Alzheimer’s disease who have neuropsychiatric symptoms, though this remains speculative and has not been prospectively evaluated in existing clinical research trials to date. Unfortunately, as with many supplements that are not FDA-regulated, advertising language for GBE products is often unfounded and uses imprecise terms, for example claiming to “support” or “promote” memory and cognitive function, terminology that can easily be mistaken as claiming clinical efficacy for memory enhancement [73,74,75].

### 3.3. Curcumin (Turmeric)

Curcumin is a polyphenolic compound contained in the widely used curry spice turmeric, derived from the rhizome (root) of the herb Curcuma longa [76]. Curcumin accounts for around 5% of turmeric, giving the spice its color rather than flavor. It is also a commonly used substance in traditional Ayurvedic medicine. It has demonstrated in vitro antioxidant and anti-inflammatory properties and has thus been suggested for a plethora of inflammatory conditions, including cognitive decline and dementia.

#### 3.3.1. Efficacy Data

Anecdotally, Alzheimer’s disease has been found to have a lower prevalence in India as compared to the United States [77], and at least one study suggested that healthy older people who consume curry more frequently have better cognitive performances [41]. Animal studies reported reduced levels of amyloid plaques, oxidative stress, and cognitive deficits associated with exposure to curcumin [43,44]. In vitro evidence supports similar conclusions [45].

Human reports on effects of curcumin use are limited. Several small, short, randomized clinical trials have shown negative results. Baum et al. conducted a six-month double-blind clinical trial of 34 patients with probable or possible AD and found no difference between drug and placebo on MMSE scores, though notably there was no cognitive decline in the placebo group in this short trial [36]. Another 24-week double-blind study of 36 patients with mild to moderate AD showed no difference between drug and placebo groups on cognitive tests, on their rate or severity of neuropsychiatric symptoms, or their ability to perform activities of daily living [37]. In a non-controlled case series, Hishikawa et al. reported three patients with Alzheimer’s disease who had improvements in neuropsychiatric symptoms after 12 weeks of treatment, and one of those patients had a five-point improvement on the MMSE [42].

In an 18-month double-blind, placebo-controlled trial of curcumin in healthy adults, Small et al. reported statistically significant improvements in memory and attention, in addition to decreased binding in the amygdala on FDDNP-PET, a measure of brain accumulation of Alzheimer’s pathological changes [39]. Of note, there is evidence that curcumin has overall low bioavailability; thus, Small et al. and other more recent trials have been investigating more bioavailable, lipidated, and other forms, with mixed results, though notably, these studies continue to be small and frequently focused on healthy individuals [38,40,46].

#### 3.3.2. Safety Data and Side Effects

Curcumin is generally considered safe, though the studies discussed above commonly revealed several participants with gastrointestinal side effects such as pain, gastritis, and nausea. Some animal studies have noted increased risks of conditions such as hepatotoxicity, thyroid follicular cell hyperplasia, lung cancer, gastric ulceration, iron deficiency, and cellular hyperplasia at very high doses, though it is unclear how significant this is for typical supplement doses [78]. Curcumin is noted in a recent safety review of drug-induced liver injury related to supplements, although the degree of risk is uncertain [79,80]. There are no data to suggest a carcinogenic effect in humans at typical doses of 1200 mg/day or less.

#### 3.3.3. Summary

Though epidemiological and in vitro mechanistic support for potential efficacy of curcumin exists, robust clinical data for its efficacy in cognitive impairment is lacking, and small preliminary studies all appear negative. Issues with absorption and bioavailability may account for some of this discrepancy, but attempts to correct this have so far not proven clearly successful. Similarly to Gingko biloba, advertising language unfortunately continues to mention unfounded and non-specific claims such as “cognitive support” [81].

### 3.4. Neuriva^®^

Neuriva^®^ (Reckit Benckiser, Slough, UK) is marketed as “a science-backed dietary supplement and complementary digital training and support program” since 2019 by RB (Reckitt Benckiser LPC)” [82]. In its initial formulation, Neuriva included two ingredients—“NeuroFactor” (made from coffee cherry extract) and plant-sourced phosphatidylserine (PS). Neuriva now has several formulations: Neuriva Original, Neuriva Plus, and Neuriva Ultra (Table 2).

The website reports that NeuroFactor^®^ is made of coffee cherry extract, which is “rich in polyphenols that support brain and overall health”. Phosphatidylserine (PS) is a lipid and major component of all cell membranes, including neurons and the myelin sheath [83].

#### 3.4.1. Efficacy Data

An in vitro study of Neuriva^®^ and other “brain supplements” reported a non-zero association between Neuriva and interaction with Aβ 1-42 fibrils and tau protein paired helical/straight filaments. [84] Another study from 2013 by Applied BioClinical, Inc. measured fasting serum levels of brain-derived neurotrophic factor (BDNF) in healthy individuals before and after supplementation with whole coffee fruit concentrate powder, green coffee caffeine powder, grape seed extract powder, and green coffee bean extract powder. Those given whole coffee fruit concentration powder (*n* = 10) had 143% increase in serum BDNF levels after 2 h [85].

There is limited information available on supplementation with the other component, phosphatidylserine (PS), in humans. PS is an acidic phospholipid made up of essential fatty acids, which play a role in determining neuronal membrane surface potential and the local ionic environment that facilitates action potential propagation and normal neuronal activity [83,86]. In the cortex of the human brain, a reduction in the DHA content of PS has been associated with the progression of mild cognitive impairment to Alzheimer’s dementia [87]. One scientific review of 127 papers on phosphatidylserine from 2015 found that exogenous PS (300–800 mg/d) is absorbed efficiently in humans, crosses the blood–brain barrier, and safely slows, halts, or reverses biochemical alterations and structural deterioration in nerve cells [83].

The only PubMed indexed clinical trials report on Neuriva^®^ is a randomized, double-blind, placebo-controlled study of 138 healthy adults with self-reported memory concerns who were aged 40–65 years to examine the effects of Neuriva^®^ supplementation on plasma BDNF and on a computerized cognitive assessment battery over 42 days [88]. The study took place in a single non-academic center. There was no significant difference in plasma BDNF and self-reported memory assessment. The authors report results from the cognitive assessment through numerous comparisons, but do not report how the global assessment score changed or how the multiple comparisons were statistically controlled for. The supplement was well tolerated with no significant adverse events, with the only reported AE that differed statistically from placebo being dry mouth. This trial was not registered on clinicaltrials.gov, and a search of that database does not identify any other protocols with Neuriva^®^.

With regards to the other components of Neuriva^®^ formulations, B-vitamins have been studied for decades in relation to cognition and brain health. Discussed in more detail below, the evidence from randomized clinical trials has not shown that years of vitamin B12 supplementation alone or with folic acid, vitamin B6, or both improves cognitive function in older adults with or without dementia, mild cognitive impairment, or Alzheimer’s disease [47]. “Cognivive” appears to not yet be trademarked by Neuriva but is a term only found for their Neuriva Ultra supplement [89]. It is an Alpina galanga extract is part of the Zingiberaceae family, which also includes curcuma (curcuma root (turmeric)) and zingiber (ginger root) [90]. A galanga is commonly used as a culinary herb in Asia, especially Indonesia and Thailand. It has also been used in Ayurvedic medicine for its high antioxidant content [91]. One randomized clinical trial showed that A galanga increased mental alertness 1, 3, and 5 h after supplementation, while the combination of A galanga with caffeine prevented caffeine crash and improved sustained attention at 3 h [92].

#### 3.4.2. Safety Data and Side Effects

The single clinical trial, which was funded by RB and not registered on clinicaltrials.gov, reported that 42 days of exposure to Neuriva^®^ supplementation was “safe [and] well tolerated” [88]. No other published clinical safety data exist.

#### 3.4.3. Summary

There are few available data to derive the efficacy or safety of the supplements marketed under the Neuriva^®^ masthead. They have not been investigated by a rigorous scientific trial either for efficacy or for safety. B-vitamins are discussed in more detail below.

### 3.5. B-Vitamins

B-vitamins are water-soluble molecules that act as important cofactors to many biological pathways. They are naturally occurring in most plant-based and animal products, and most processed foods manufactured in the U.S. are fortified with high concentrations of B-vitamins [47]. B-vitamins, especially B12 and folate, have been proven to lower homocysteine levels which, when elevated, can lead to endothelial dysfunction and brain damage as seen by white matter hyperintensities [93,94]. Elevated homocysteine levels have been suggested to be independent risk factors for dementia [95,96]. It is hypothesized that elevated homocysteine levels might exert a negative effect on the brain via the cerebrovascular system (i.e., increasing risk of microvascular damage and vascular dementia), activation of tau kinases leading to neurofibrillary tangle deposition, and inhibition of methylation reactions [96]. Changes in basal and postmethionine load concentrations of total homocysteine and cystathionine after B-vitamin intervention have been demonstrated [97,98]. Whether B-vitamin supplementation can prevent or slow down cognitive decline via lowering of homocysteine or by other methods, however, has remained an open question [99]. Overall, findings are mixed for whether B-vitamin supplementation can enhance cognitive function or prevent dementia [93].

**Pyridoxine (Vitamin B6)** is a cofactor involved in over 100 enzyme reactions, including protein, carbohydrate, and lipid metabolism [90,93]. Vitamin B6 also plays a role in cognitive development through the biosynthesis of neurotransmitters and in maintaining normal levels of amino acids in the blood [100,101]. Vitamin B6 deficiency is associated with microcytic anemia, electroencephalographic abnormalities, dermatitis with cheilosis (scaling on the lips and cracks at the corners of the mouth) and glossitis (swollen tongue), depression and confusion, and weakened immune function [90,100,101].

Several studies have demonstrated an association between vitamin B6 and cognitive function in the elderly [90,93,100,101,102]. The Boston Normative Aging Study found an association between higher serum vitamin B6 concentrations and better memory test scores in a group of 70 men aged 54–81 years [49]. A Cochrane Review found no evidence that short-term vitamin B6 supplementation (for 5–12 weeks) improves cognitive function or mood [51], and a meta-analysis of various B-vitamins found no difference in incident dementia between relatively high and low intake of B6 (total hazard ratio of 0.95 with 95% confidence interval of 0.73–1.22 over five studies) [99].

**Folate (Vitamin B9)** is a water-soluble vitamin found in dark leafy greens, nuts, and legumes [89]. Some, but not all, observational studies have reported association between low serum folate concentrations and both poor cognitive function and higher risk of dementia and Alzheimer’s disease [47,52,93,96].

While there is a possible association between nutritional deficiency and incident cognitive decline, clinical trials of folic acid supplementation have not demonstrated an effect on cognitive function or in reducing the risk of dementia or Alzheimer’s disease. In one randomized, double-blind, placebo-controlled trial in the Netherlands, 195 people age 70 years or older with no or moderate cognitive impairment received 400 mcg folic acid + 1 mg vitamin B12; 1 mg vitamin B12 only; or placebo for 24 weeks [54]. Treatment with folic acid plus vitamin B12 reduced homocysteine concentrations by 36% but did not have an impact on cognitive function. A 2024 study of 12,025 participants having no neurological symptoms reported that increased folate intake was associated with more connectivity in several neural networks implicated in AD, including the primary visual, visuocerebellar, cerebello–thalamo–cortical, and the posterior default mode networks [93]. Interestingly, vitamins B12 and B6 were independently associated with less functional connectivity in several other networks [93].

As part of the Women’s Antioxidant and Folic Acid Cardiovascular Study, 2009 U.S. women aged 65 years or older at high risk of cardiovascular disease were randomly assigned to receive daily supplements containing 2500 mcg folic acid + 1 mg vitamin B12 and 50 mg vitamin B6 or placebo [50]. After an average of 1.2 years, B-vitamin supplementation did not affect mean cognitive change from baseline compared with placebo. However, in a subset of women with a low baseline dietary intake of B-vitamins, supplementation with these B-vitamins appeared to be beneficial.

**Cyanocobalamin (Vitamin B12)** is found in foods of animal origin, including fish, meat, poultry, eggs, and dairy products [47]. It is required for the development, myelination, and function of the central nervous system; healthy red blood cell formation; and DNA synthesis [90]. Because this vitamin contains the mineral cobalt, compounds with vitamin B12 activity are collectively called “cobalamins”. Several observational studies have found correlations between low serum vitamin B12 concentrations alone or in combination with high folate concentrations and poor cognitive function [47,53]. For example, analysis of cross-sectional 2011–2014 NHANES data on 2420 adults aged 60 years or older found that low vitamin B12 levels (MMA greater than 0.27 micromol/L or serum vitamin B12 less than 203 pg/mL (150 pmol/L)) and high folic acid levels—unmetabolized serum folic acid greater than 0.44 mcg/L (1 nmol/L) or serum total folate higher than 32.7 mcg/L (74.1 nmol/L)—were associated with an almost two-to-three times higher risk of cognitive impairment [53].

In the absence of nutritional deficiency, evidence from randomized trials has not demonstrated that vitamin B12 supplementation for 1 to 2 years improves cognitive function in older adults with or without dementia, mild cognitive impairment, or Alzheimer’s disease [47,93]. Supplements had no effect on global cognitive function, even when administered for up to 5 years and appeared to have no impact when administered for 5 to 10 years [48].

Similarly, supplementation with vitamin B12, alone or with other B-vitamins, does not appear to decrease the risk or slow the progression of dementia or Alzheimer’s disease in older adults. A Cochrane Review evaluated the effects of vitamin and mineral supplements on cognitive function and dementia in people with mild cognitive impairment [103]. The review included five trials with 879 participants that investigated B-vitamin supplements (one study of folic acid only and four trials of vitamins B6, folate, and B12). Study duration ranged from 6 to 24 months, and there was no meaningful or significant effect on episodic memory, executive function, speed of processing, or quality of life. A follow-up Cochrane Review from 2003 included all randomized, double-blind trials in which vitamin B12 was compared to placebo and found no significant evidence of a treatment effect on cognitive function in patients with reported cognitive impairment [55].

#### 3.5.1. Safety Data and Side Effects

B-vitamins are naturally occurring in plant-based and animal foods and fortify U.S. cereals, grains, and desserts. Most B-vitamins are not associated with known toxicities. Niacin (vitamin B3) commonly causes flushing and GI side-effects and, at very high doses, has been associated with liver injury and myopathy. High doses of pyridoxine (vitamin B6) have been related to severe and progressive sensory neuropathy characterized by pain, falls, and ataxia. Symptom severity appears to be dose dependent (1–6 g/day), and the symptoms usually stop if the patient discontinues the pyridoxine supplements as soon as neurologic symptoms appear [104]. High doses of folic acid have been associated with progression of pre-neoplastic lesions, possibly increasing the risk of colorectal or other cancers in certain individuals [105]. Additionally, elevated folic acid intake may mask megaloblastic anemia caused by vitamin B12 deficiency, leading to irreversible neurologic damage in the form of subacute combined degeneration of the spine [47]. Due to possible adverse effects from excessive supplementation, an upper limit of supplemental folate has been established—this varies by sex and age and is available online [47]. Vitamin B12 has not been associated with an identified toxicity.

#### 3.5.2. Recommendations

Several large reviews have evaluated the effect of B-vitamins on cognitive function. Most of the authors concluded that in the absence of nutritional deficiency, supplementation with folic acid alone or in combination with vitamins B12 or B6 does not improve cognitive function in individuals with or without cognitive impairment [102]. This aligns with the American Academy of Neurology (AAN) Practice Parameter for evaluation of vitamin B12 levels in patients presenting with memory complaints [106]. In the absence of nutritional deficiency, we generally do not recommend high-dose B-vitamin supplementation in our practice.

### 3.6. Multivitamins

Multivitamins are a heterogenous group of supplements usually consisting of combinations of nutrients and vitamins, some of which may be in doses above general daily recommendations. While many of the vitamins included in such supplements are necessary for physiologic health and, when deficient, lead to disease (i.e., vitamin C deficiency and scurvy or niacin deficiency and pellagra), data to support the regular use of these supplements for general health and for cognition in people with normal diets and normal intestinal absorption are limited.

#### 3.6.1. Efficacy Data

The cognitive substudies embedded in the Age-Related Eye Disease Studies (AREDS) demonstrated consistent findings across these two well-characterized North American cohorts [107,108]. The authors reported that nutrient-rich dietary patterns were protective against cognitive impairment, though they did not demonstrate slower cognitive decline in individuals. Importantly, the authors noted “for each nutrient, the likelihood of cognitive impairment changed over time in a similar way, irrespective of nutrient intake quintiles; similarly, the rate of change in cognitive function scores did not differ. Hence, cognitive decline did not appear to be significantly faster or slower with higher intake of any one of the nutrients analyzed” [108]. The authors concluded their findings were consistent with existing reports that find association between greater adherence to a Mediterranean-style diet and protection against cognitive decline [107], but did not find evidence to support additional supplementation of any of the nutrients studied. Similarly, the Cocoa Supplement and Multivitamin Outcomes Study for the Mind (COSMOS-Mind) did not detect a protective effect against incident cognitive decline (progression to MCI or dementia) for people taking multivitamins versus placebo over three years [109], although a post-hoc analysis did report that those taking multivitamins showed better performance on in-person cognitive tests over a two-year observation window [56].

#### 3.6.2. Safety Data and Side Effects

A multi-cohort analysis of data from 3 large studies that followed 390,124 generally healthy adults followed for over 20 years found there was no benefit or detriment to mortality for people regularly taking multivitamins compared to those who did not [110].

#### 3.6.3. Summary

Taken together, these data suggest no particular benefit or harm associated with multivitamin use in the general population. An important caveat is that patients having nutrient-poor diets and/or documented vitamin deficiencies may stand to benefit from multivitamin supplements. Our practice is to counsel patients that in the absence of a demonstrated vitamin deficiency, no evidence exists that supports high-dose supplementation of specific vitamins as helpful in slowing or preventing cognitive decline. Multivitamin use is unlikely to be harmful and is likely sufficient for people who have concerns about micronutrient intake. We recommend that patients follow a Mediterranean-style or DASH diet (Dietary Approaches to Stop Hypertension), both of which focus on a whole-food diet rich in fruits and vegetables with limited processed foods. We emphasize that both diet and regular physical exercise have well-documented associations with better cognitive outcomes.

## 4. Discussion

Dietary supplements are used by a high proportion of older adults in the U.S., with many citing cognitive concerns (whether for the prevention or treatment of existing symptoms) as their motivation. As outlined in this review, patients’ desire to forestall cognitive decline is met by the supplement industry with ever-increasing offerings based on preliminary, limited, and/or incomplete data, and the lack of regulation of the supplement industry allows for advertising language that implies much more benefit than the data would support (Table 1).

While this review does not aim to provide a comprehensive analysis of all supplements marketed or used for cognition, the ones examined are a group of supplements actively being used by our patients, and they are representative of the general trends in the industry. To our knowledge, no dietary supplement has a significantly better evidence base for the prevention or treatment of cognitive decline than the supplements detailed in this review.

Several issues exist, besides the lack of strong efficacy data. Two recent reviews emphasize the unknown scope of unknown compounds in dietary supplements [111,112]. These may be introduced as intentional undeclared ingredients (a 2001–2002 survey of bodybuilding supplements in 13 countries found 15% contained undeclared anabolic steroids [113]) or as unintentional contaminants related to quality control issues during manufacturing, processing, or packaging. Still others may include labeled ingredients listed using confusing nomenclature that obscures the identity of the substance. As Jędrejko et al. outline, this presents challenges to regulatory and athletic authorities [111]. It also presents a challenge to consumers and physicians who may be unaware of interactions or risks associated with dietary supplements. Highlighting this, a recent review estimated the proportion of drug-induced liver injuries due to dietary supplements is at around 20%. Bodybuilding and weight loss supplements account for almost half of these injuries [114].

In an effort to serve U.S. consumers, in 2013, the NIH established the Dietary Supplement Label Database (https://ods.od.nih.gov/Research/Dietary_Supplement_Label_Database.aspx, accessed on 15 November 2024), which “catalogs all information printed on labels of dietary supplement products sold in the United States” [89]. This searchable database is regularly updated and maintained by the NIH. Unfortunately, the scope of the challenge presented by misleading advertising practices, constantly evolving trends in and predilections for new dietary supplements, and (sometimes intentionally) complex labels diminish the practical value of this resource for patients and clinicians alike. To wit, as of this writing, the database has already catalogued over 192,000 labels.

While concerns over the use and safety of supplements have been highlighted by numerous clinicians [115,116], scientists [117], and patient advocacy groups [118], the focus is often on the lack of proven efficacy, deceptive marketing, and potential for side effects. There has been less focus on the cost borne by patients. One reason for this is that many herbs, supplements, and “medical foods” are chosen for their relatively low cost, at least as compared to potentially promising treatments such as monoclonal antibodies, which are orders of magnitude more expensive. That being said, economic impact is important to examine in an industry whose market size is expected to reach USD 13.38 billion worldwide by 2028 on an annual growth rate of up to eight percent [1,119], especially when the population paying this cost is often elderly and may be on a fixed income or living off of retirement savings.

There are limitations to the data and reports available for a review of this topic. The lack of regulation of nutritional supplements results in variable quality control regarding the content and quantity of individual ingredients in commercially available supplements. Further, it is plausible that existing randomized clinical trials may have investigated doses of supplements that were too small to have demonstrated an effect. Taken together, these limitations identify a need for further clinical research of dietary supplements in people having dementia.

To our knowledge, rigorous data do not exist regarding supplements’ cost relative to their benefit and the overall economic impact at the individual level. Understandably, such studies are difficult to carry out for many reasons, including the wide array of potential supplements and supplement retailers, the varied socioeconomic status of the target population, and the fact that supplements are not always reported in medication lists and medical histories, to name a few. To be sure, the cost of all the supplements discussed in this review pale in comparison to some conventional, FDA-approved treatments, especially in the age of monoclonal antibody therapy. However, in our experience and based on the survey discussed in the methods, patients who take supplements for any indication often take more than one supplement. Thus, while the cost of any single supplement may be negligible for some, for those taking multiple supplements and/or for those on fixed incomes or with a low disposal income, these costs can add up. Indeed, in our survey, the range of monthly expenditure for all dietary supplements was from USD 0 to USD 300, with a mean of USD 43.71 and a median of USD 20. Additionally, 17% reported that the amount of money spent on supplements is at least sometimes burdensome or difficult to afford. Ultimately, more rigorous research is required to determine the true range of the economic impact of supplement use.

The supplement industry is not regulated by the FDA, but rather by the Federal Trade Commission (FTC). Although the FTC produced and updated in 2022 its compliance guidance for health products [120], the scope of this document speaks to the challenging complexity of monitoring and policing this industry. The guidance cites a number of complaints the FTC has successfully prosecuted in relation to false advertising, but the document’s length suggests the supplement industry has a long history of supporting numerous small, unregistered trials with subsequent reports of “positive results” and no reports of the more numerous negative results or of results counter to the desired advertising claims [121,122,123].

## 5. Conclusions

Our aim in this narrative review was to examine the supporting use of six commonly used supplements reported in a survey of our clinic. Little or no evidence is available to support the use of any of these substances to ameliorate memory loss or other cognitive symptoms. Although most would appear safe, the unknown rates of inclusion of cholimimetic, stimulant, and/or other psychopharmacological compounds as contaminants must give some pause when patients are consuming these over-the-counter substances. Further research is needed to understand the long-term effects of dietary supplements and to consider the implications of their potential costs, which should not be ignored when products are targeted toward vulnerable populations.

## Figures and Tables

**Table 1:** Summary of reviewed data by study design.

Supplement	Research Reviewed by Study Design Authors (Year)							
	Double-Blind Placebo-Controlled	Randomized Trial	Cochrane Review	Cohort or Longitudinal	Case Study	Association, Cross-Sectional	In Vitro	Animal Models
Apoaequorin	Moran et al. (2016) [11]						Detert et al. (2013) [12]	Ehlers et al. (2020) [11]
Ginkgo biloba	Schneider et al. (2005) [13] Vellas et al. (2012) [14] Gavrilova et al. (2014) [15]	DeKosky et al. (2008) [16] Snitz et al. (2009) [17] Ihl et al. (2012) [18] Li et al. (2017) [19]	Birks et al. (2009) [20]				Amri et al. (1996, 2002) [21,22] Oyama et al. (1996) [23] Zhu et al. (1997) [24] Klein et al. (1997) [25] Bastianetto et al. (2000) [26] Zhou et al. (2000) [27] Song et al. (2000) [28] Yao et al. (2004) [29] Longpre et al. (2006) [30] Gargouri et al. (2018) [31]	Schindowski et al. (2001) [32] Drieu et al. (2000) [33] Wu et al. (2006) [34] Tunali-Akbay et al. (2007) [35]
Curcumin	Baum et al. (2008) [36] Ringman et al. (2012) [37] Rainey-Smith et al. (2016) [38] Small et al. (2018) [39] Cox et al. (2020) [40]			Ng et al. (2006) [41]	Hishikawa et al. (2012) [42]			Frautschy et al. (2001) [43] Lim et al. (2001) [44] Yang et al. (2005) [45] Begum et al. (2008) [46]
Neuriva	Doma et al. (2023) [47]							
B-complex			Rutjes et al. (2018) [48]			Riggs et al. (1996) [49]		
Vitamin B6	Kang et al. (2008) [50]		Malouf et al. (2003) [51]			Riggs et al. (1996) [49]		
Vitamin B9	Kang et al. (2008) [50]		Malouf et al. (2008) [52]			Riggs et al. (1996) [49] Bailey et al. (2020) [53]		
Vitamin B12	Kang et al. (2008) [50]		Eussen et al. (2006) [54] Malouf et al. (2003, 2008) [52,55]			Riggs et al. (1996) [49] Bailey et al. (2020) [53]		
Multivitamin		Sachs et al. (2023) [56]						

**Table 2:** Reported ingredients of several formulations of Neuriva^®^ [83].

Neuriva^®^ Original	Neuriva^®^ Plus	Neuriva^®^ Ultra
NeuroFactor™	NeuroFactor™	NeuroFactor™
Phosphatidylserine (PS)	Phosphatidylserine (PS) Vitamin B6 (pyridoxine)	Phosphatidylserine (PS) Vitamin B6 (pyridoxine)
	Vitamin B9 (folate)	Vitamin B9 (folate)
	Vitamin B12 (cyanocobalamin)	Vitamin B12 (cyanocobalamin) Cognivive (alpina galanga extract)

## Data Availability

No new data were created for this review.
